# Cross-Modal Plasticity Results in Increased Inhibition in Primary Auditory Cortical Areas

**DOI:** 10.1155/2013/530651

**Published:** 2013-10-31

**Authors:** Yu-Ting Mao, Sarah L. Pallas

**Affiliations:** ^1^Department of Biology, Georgia State University, Atlanta, GA 30303, USA; ^2^Neuroscience Institute, Georgia State University, P.O. Box 5030, Atlanta, GA 30302-5030, USA

## Abstract

Loss of sensory input from peripheral organ damage, sensory deprivation, or brain damage can result in adaptive or maladaptive changes in sensory cortex. In previous research, we found that auditory cortical tuning and tonotopy were impaired by cross-modal invasion of visual inputs. Sensory deprivation is typically associated with a loss of inhibition. To determine whether inhibitory plasticity is responsible for this process, we measured pre- and postsynaptic changes in inhibitory connectivity in ferret auditory cortex (AC) after cross-modal plasticity. We found that blocking GABA_A_ receptors increased responsiveness and broadened sound frequency tuning in the cross-modal group more than in the normal group. Furthermore, expression levels of glutamic acid decarboxylase (GAD) protein were increased in the cross-modal group. We also found that blocking inhibition unmasked visual responses of some auditory neurons in cross-modal AC. Overall, our data suggest a role for increased inhibition in reducing the effectiveness of the abnormal visual inputs and argue that decreased inhibition is not responsible for compromised auditory cortical function after cross-modal invasion. Our findings imply that inhibitory plasticity may play a role in reorganizing sensory cortex after cross-modal invasion, suggesting clinical strategies for recovery after brain injury or sensory deprivation.

## 1. Introduction

Loss of sensory drive as a result of deprivation or deafferentation can lead to a compensatory plastic reorganization of the affected sensory cortex. For example, a homeostatic downregulation of inhibition makes cortical neurons more sensitive to any remaining inputs (see [1], for review). Although the plastic response to the loss of drive can be limited to a single modality, sprouting of inputs responding to other sensory modalities into the deafferented area often results in cross-modal plasticity. For example, in deaf and blind subjects, the spared sensory cortex can be taken over by sensory inputs from other sensory modalities [2–4]. Such cross-modal inputs replace the lost inputs to some extent; thus the mechanisms of recovery might be different from recovery from manipulations affecting a single modality [5]. Because sensory inputs have been changed rather than lost entirely, the loss of inhibition seen after unimodal deprivation may be mitigated. It is important to understand whether cross-modal plasticity has similar or different effects on inhibition than within-modality plasticity because of the prevalence of cross-modal plasticity in patients suffering from deafness and blindness [3, 6, 7]. 

 Cross-modal plasticity in auditory cortex, in addition to providing new capabilities (see [8], for review), can have negative effects on the original auditory function during rehabilitation ([9, 10], see [11], for review). In prelingually deaf children with cochlear implants, auditory cortex responds poorly to auditory signals because it has been permanently taken over by cross-modal inputs [12]. As a result of this maladaptive cross-modal plasticity, deaf patients with cochlear implants exhibit poorer performance in auditory speech recognition tasks than hearing controls [9, 13]. Whether this deficit might result in part from a loss of inhibitory shaping of responses, as seen in deafness without cross-modal plasticity (see [14], for review, [15]), has not been examined.

**Figure 1:**
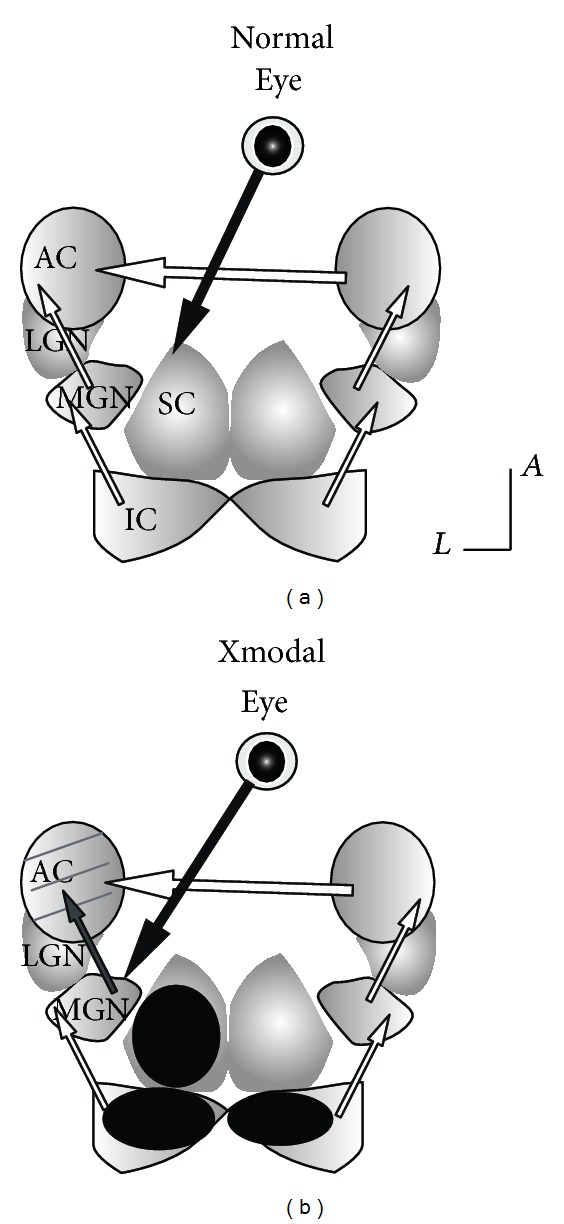
Neonatal surgery results in visual invasion to auditory pathway. (a) Normal connectivity pattern. LGN: lateral geniculate nucleus; MGN: medial geniculate nucleus; AC: auditory cortex. SC: superior colliculus; IC: inferior colliculus. Visual projection is labeled in black. Auditory projection is labeled in white. (b) Cross-modal connectivity pattern. Dark circles on inferiror colliculi and left superior colliculus represent neonatal lesion. Retinal projection was rewired to MGN. Because of residual auditory inputs from inferior colliculi and visual rewiring, the thalamocortical projection from MGN to auditory cortex was changed from white to gray to present combination of auditory and visual processing. Contralateral AC is another important source of auditory projection. Gray lines in AC of cross-modal indicate multisensory responses. *A*: anterior. *L*: lateral.

 We addressed this issue using a ferret model in which the extent of cross-modal visual invasion of the auditory pathway can be manipulated (Figure 1). Neonatal lesion of visual and auditory midbrain of ferret pups is sufficient to divert retinal projections to adjacent, deafferented auditory thalamus (MGN), which then carries visual information to auditory cortex [16–18]. In our previous work with this model, we found that primary auditory cortical areas of ferrets rewired with ectopic visual inputs (cross-modal auditory cortex, abbreviated as XMAC) retain substantial auditory function, but sound frequency tuning and tonotopy are compromised [19, 20] much as can occur in humans with peripheral hearing loss [21]. Here we examine whether inhibitory plasticity is a cause of the impaired auditory function. On the one hand, lateral inhibition could be decreased homeostatically in order to compensate for the loss of sensory drive. On the other hand, an increase in inhibition might serve to facilitate parallel processing of both auditory and visual functions in the auditory cortex of the cross-modal animals. Reciprocal inhibition may suppress responses to the less salient stimulus modality. Here we tested these hypotheses using electrophysiological, pharmacological, immunohistochemical, and immunoblot methods. We found that the change in auditory responses after acute blockade of GABA_A_ receptors was larger in the cross-modal group than in the normal group. In addition, the expression of GAD was higher in the cross-modal group than in the normal group. Our results demonstrate that inhibition is increased in auditory cortex after invasion by ectopic visual inputs. We also found that blocking inhibition could unmask visual responses in auditory cortex, suggesting that subthreshold visual responses are suppressed by strong inhibition. Facilitative or suppressive multisensory integration was not revealed by the removal of inhibition, however. Our results provide important insight into the origin of maladaptive changes that can occur with cross-modal plasticity and stress the importance of designing rehabilitative strategies in the context of the inhibitory plasticity that occurs during recovery from brain damage.

## 2. Materials and Methods 

### 2.1. Animals

In total, 27 adult pigmented ferrets (*Mustela putorius furo*) were either purchased from Marshall Farms (North Rose, NY) or bred in house. Nonlactating adults were fed Marshall Farms ferret diet and kept on a 12/12 light/dark cycle. Twelve ferrets were used exclusively for electrophysiological recording (7 in the normal group, 5 in the experimental group), 8 were used for immunohistochemical experiments (5 in the normal group, 3 in the experimental group), and 7 were used for western blotting experiments (3 in the normal group, 4 in the experimental group). All animal protocols were approved by the Institutional Animal Care and Use Committee (IACUC) at Georgia State University and met or exceeded standards of care established by the USDA and the Society for Neuroscience. 

### 2.2. Neonatal Surgery

Surgical procedures were similar to those described previously [19, 20]. Under sterile conditions, ferret kits were deeply anesthetized with isoflurane (1–4% prn) within 24 hr of birth. After the brain was exposed, the left superficial layer of the superior colliculus (sSC) and the central nucleus of the inferior colliculi (ICc) on both sides were cauterized. The brachium of the left inferior colliculus (IC) was sectioned (Figure 1). The incision was closed with surgical adhesive (VetBond, 3 M, St. Paul, MN). Subcutaneous fluids and a respiratory stimulant (doxapram, 2 mg/kg, SQ) were given postoperatively, and the kits were kept warm while they recovered from anesthesia. They were returned to their mother for rearing and maintained until adulthood, at which point they were subjected to electrophysiological recordings.

### 2.3. Preparation for Adult Electrophysiology

After the lesioned kits attained adulthood (4 months of age and above), they were prepared for terminal electrophysiology recording as described previously [19]. Unlesioned adult animals served as a control for the effects of surgery. The ear canal of each ferret was examined before surgery with an otoscope. Animals were given atropine (0.4 mg/kg SQ) and doxapram (2 mg/kg, SQ) before anesthesia to counteract bradycardia and to reduce mucosal secretions. Anesthesia was induced by intramuscular injection of ketamine (40 mg/kg, IM) and diazepam (2 mg/kg, IM). Dexamethasone (1 mg/kg, IM) was given every 24 hours to prevent cerebral edema. After the cephalic or femoral vein was cannulated, a surgical plane of anesthesia was maintained with a continuous infusion (2–5 mL/h, IV) of a mixture of dexmedetomidine (0.022 mg/kg/hr) and ketamine (5 mg/kg/hr) in lactated Ringer's with 5% dextrose [22, 23]. Atropine (0.4 mg/kg, SQ) was given as necessary to counteract the bradycardia caused by dexmedetomidine. A tracheotomy was performed for artificial ventilation (SAR 830/P ventilator, CWE Inc., Ardmore, PA). Body temperature was maintained at 38°C with a warming pad. Vital signs including EKG, respiration rate, muscle tone, withdrawal reflexes, and end-tidal CO_2_ were monitored during the entire process. The eyes were kept moist with commercial artificial tears solution. Animals were placed in a stereotaxic device to stabilize the head. After the skin on the top of head was incised by a scalpel, the temporal muscles were retracted bilaterally from the skull. Optic chiasm stimulation was used to evoke reliable and consistent activation of visual inputs. Two burr holes (at coordinates A5.5 ± L1.5) were drilled for optic chiasm recording/stimulation electrodes. Saline was added around the drilling area frequently to prevent overheating. Two tungsten rods with Teflon insulation (0.008 bare, 0.011 coated, A-M systems, Inc., Carlsborg, WA) were lowered to a depth (8–10 mm) that yielded strong visual responses to a strobe light. The tungsten rods were connected to a preamplifier and then switched to connect to a stimulus isolation unit (BAK Electronics, Mount Airy, MD). A 0.8–1.0 cm diameter craniotomy was drilled over the left auditory cortex. The dura was removed, and auditory cortex was covered with sterile saline. A metal bar was cemented on the contralateral (right) side of the skull to stabilize the head. The right ear bar was then released to allow access for auditory stimulation. 

### 2.4. Acoustic Stimuli and Visual Stimuli

Acoustic stimuli were generated by TDT system III hard- and software (Tucker-Davis Technologies, Alachua, FL). A calibrated earphone (ER-2 insert earphone, Etymotic Research, IL) was placed in the pinna at the entrance to the right ear canal. All auditory stimuli were given contralaterally. White noise bursts (5 ms ramp, 40–100 ms duration, 80 dB SPL) were used to search for sound-responsive units. After a responsive neuron was found, pure tones were given in a pseudorandom order ranging in 2 kHz steps from 2 kHz to 18 kHz or ranging in half octave steps from 500 Hz to 16 kHz with an intensity of 30 to 80 dB SPL (50 ms duration, 5 ms ramp). Bipolar electrical stimulation of the optic chiasm was also applied (single pulses at 0.5–1 mA, 60 *μ*s duration) to test whether the units were multisensory neurons. In addition, light stimuli were presented on a computer screen ~40 cm from the eyes. Moving bars, gratings, and flashes were used to elicit responses. After a visual neuron was found, the computer screen was moved to a location and height that aligned the center of the visual receptive field approximately at the center of the screen. As reported previously [19, 24], responses to light are difficult to evoke from the cross-modal ferret auditory cortex because the animals are anesthetized, the retinal ganglion cells that rewire their projections to the auditory pathway arise from W-cells, and the visual inputs are sparse. We did not focus our recordings in the lateral AC where subthreshold visual responses have been found in normal ferrets [25] but instead spaced our recordings throughout the region.

### 2.5. Multibarrel Electrodes for Recording and Iontophoresis

Three-barrel glass micropipette blanks (A-M Systems Inc., Sequim, WA) were used to record evoked activity and for application of drugs. The blanks were pulled by a vertical puller (Kopf vertical pipette puller 720, David Kopf instruments, Tujunga, CA). The tip was examined under a microscope before recording. Any electrodes with a tip bigger than 15 *μ*m were discarded. The recording barrel was filled with 3 M NaCl. A silver wire was inserted into the recording barrel to connect it with the preamplifier. The other two barrels were used as ionotophoresis barrels and were filled with 3 mM gabazine (SR-95531, Sigma-Aldrich, St. Louis, MO, pH 3.0). The ionotophoresis barrels were connected to the headstage of a three-channel ionotophoresis device (Cygnus Technology, Inc., Delaware Water Gap, PA) via silver wire. The headstage and the preamplifier were grounded together to the skin, which served as the ground for the entire recording apparatus. A retaining current (−10 nA) was applied in each barrel to prevent drug leakage. Ejecting currents were +5~+10 nA. Application of drugs was maintained throughout the testing period. Typically it took 5 minutes for running a testing series of different sound frequencies. The electrode was advanced under the pial surface in 5 *μ*m steps up to 2000 *μ*m by a hydraulic microdrive (Kopf Instruments, Tujunga, CA). The first unit encountered in each penetration was isolated and characterized. Penetrations were limited to primary auditory cortex (A1) and anterior auditory field (AAF) as defined by sulcal landmarks and cytoarchitectonics [19, 22]. In order to avoid potential diffusion of iontophoretic drugs, penetration sites were at least ~400 *μ*m apart. Neural responses were amplified (×10000, BAK Electronics, Inc., Mount Airy, MD), bandpass filtered (500 Hz to 5 kHz), and monitored on a digital oscilloscope (Hameg Instruments, Mainhausen, Germany). Responses to 5–10 stimulus presentations were collected from each recording site and digitized at 25 kHz (Brainware software, Tucker-Davis Technologies Inc., Alachua, FL). Spontaneous activity was recorded for 50 ms before each trial. The evoked responses were averaged and normalized to the mean level of recorded spontaneous activity. The recording continued for 1-2 days, after which the animal was deeply anesthetized (>100 mg/kg sodium pentobarbital, IP) for perfusion. The perfused brain was extracted for histological examination. Brains were sectioned and stained for Nissl substance. The analysis of lesion size was performed as reported previously [19, 20].

### 2.6. Electrophysiological Data Analysis

 After recording, spike sorting was performed by Brainware software (Tucker-Davis Technologies Inc., Alachua, FL). Single units were isolated as described previously [19]. For each isolated single unit, evoked responses were defined as those within 300 ms of the stimulus onset that were above the mean level of spontaneous activity by at least 20% of the peak firing rate. The boundaries of each frequency tuning curve were defined as the stimuli (intensity and frequency) that yielded these evoked, excitatory responses [22, 26]. The tuning curves were plotted using a Matlab function kindly provided by Professor Jennifer Bizley (Ear Institute, University College of London, London, UK). The characteristic frequency (CF) of each unit was defined as the frequency at which responses were evoked at the lowest sound level. Bandwidth was determined as the width of the tuning curve at 10 dB above the minimum threshold. Multisensory units were defined either as neurons that responded both to visual and auditory stimuli or as neurons that only responded to one modality but could be significantly modulated by stimulation with the other modality [27]. Statistical significance between groups was determined by comparing the number of spikes per trial in response to both stimulus modalities, using Student's *t*-test (significance at *P* < 0.05). 

### 2.7. Immunohistochemistry

Immunohistochemical and immunoblot assays were performed exclusively on adult animals that were not used in electrophysiological experiments. To harvest tissue for immunohistochemistry, animals were given a high dose of sodium pentobarbital (>100 mg/kg, IP) to induce deep anesthesia and they were perfused through the heart with 0.1 M phosphate buffered saline (PBS, pH 7.4) followed by a mixture of 4% paraformaldehyde and 0.2% glutaraldehyde in 0.1 M phosphate buffer (PB, pH 7.4). Brains were extracted and postfixed with 4% paraformaldehyde/30% sucrose in PB for 24 to 48 hours at 4°C then transferred to 30% sucrose for another 24 to 48 hours for cryoprotection. Brains were sectioned frozen in the coronal plane, alternating between 50 *μ*m and 30 *μ*m in thickness. One set of 50 *μ*m sections at 160 *μ*m intervals was used for Nissl staining and, one set of 30 *μ*m sections at 160 *μ*m intervals was used for GABA immunohistochemistry. All sections were stored in 0.1 M PB until use. Sections were processed free-floating using standard avidin-biotin-DAB techniques. Sections were rinsed in 0.1 PBS with 0.02% sodium azide (PBS/A), 0.34% L-lysine, and 0.05% sodium periodate (NaIO_4_) for 1 hour to reduce free aldehydes. Nonspecific binding was blocked by incubation with 3% normal goat serum (NGS) in PBS/A for 1 hour at room temperature. We used a mouse monoclonal anti-GABA antibody (mouse anti-GABA Clone 5A9, ab86186, Abcam, Cambridge, UK) that has been successfully used in our lab both in ferrets and in hamsters [28, 29]. Sections were incubated with the primary antibody (diluted 1 : 1000 with PBS/A and 3% NGS) for 48 to 72 hours at 4°C. After rinsing in PBS/A, sections were incubated with the secondary antibody for 2 hours (biotinylated goat anti-mouse in PBS/A with 3% NGS, diluted 1 : 200). The secondary antibody was visualized by incubation with avidin-biotin-peroxidase complex (ABC, Vectastain ABC Elite kit, Vector laboratories, Burlingame, CA) for 1 hour at a dilution of 1 : 160. Sodium azide was left out of the buffer after incubation in the secondary antibody. The peroxidase reaction was performed with 0.01% diaminobenzidine (DAB) and 0.0002% hydrogen peroxide. Nickel ammonium sulfate (1%) and 0.34% imidazole were added to the solution to intensify the reaction product. To control for the specificity of the secondary antibody the primary antibody was omitted from the procedure. In this case, no neuronal staining was found. Sections were mounted, dehydrated, and coverslipped with Permount (Fisher, Pittsburg, PA). Micrographs were taken of immunolabeled sections and adjacent Nissl-stained sections with a Zeiss microscope using Zeiss Axon Vision 3.1 software (Carl Zeiss MicroImaging, Thornwood, NY). 

### 2.8. Western Blotting

Animals were euthanized with sodium pentobarbital (>100 mg/kg, IP). Brains were extracted and immediately frozen in 2-methylbutane on dry ice. The brains were stored at −80°C until use. The Western blotting protocol was modified from a previous study [30]. The auditory cortex surrounded by suprasylvian sulcus was separated and homogenized in 500 *μ*L lysis buffer (10 mM EGTA, 5 mM EDTA, 1 mM DTT in 10 mM PB, pH 7.0, a protease inhibitor cocktail, and 1 mM phenylmethylsulfonyl fluoride (PMSF)). The homogenates were fractionated by centrifuging at 16,000 ×g for 10 min at 4°C. The supernatants were collected as a crude soluble fraction (for GAD Western blots) and placed on ice. The pellets were resuspended in 300 *μ*L 2 mM HEPES, pH 7.2 and ultracentrifuged at 4°C for 45 min at 200,000 ×g. The supernatants from this second spin were discarded. The pellets were resuspended in 280 *μ*L 0.5 mM HEPES, 0.32 M Sucrose pH 7.3 and centrifuged at 4°C for 8 min at 450 ×g. The supernatants were collected as a membrane fraction (for GABA receptor Western blots). Protein concentrations were measured by a plate reader (Spectramax 340PC, Molecular Devices Corporation, Sunnyvale, CA) using a standard BCA assay (BCA Protein assay kit, Thermo Scientific, Rockfold, IL). 10 *μ*g samples per lane were mixed with an equal volume of Laemmli buffer (Bio-Rad, Hercules, CA) and 5% mercaptoethanol, and were denatured at 60°C for 15 min. Samples and standards (Precision Plus protein standard, Bio-Rad) were run on an 8% polyacrylamide SDS gel at 110 V for 100 min. After electrophoresis, proteins were transferred to nitrocellulose membranes using electroblotting (Bio-Rad) at 340 mA for 90 min on ice. The membranes were then stained with Ponceau S and washed in TBST (0.05 M Tris, 0.9% NaCl, 0.1% Tween-20). Non-specific binding was blocked by incubating in 5% non-fat dry milk and 0.1% TBST for 30 min. Blots were incubated overnight with the primary antibodies in 0.1% TBST with 2% non-fat dry milk (rabbit anti-GAD65/67 1 : 10000, Millipore, Billerica, AB1511; rabbit anti- GABA_A_ *α*1 subunit 1 : 1000, AB5946, Millipore, Billerica, MA; rabbit anti- GABA_A_ *γ*2, 832-GG2C, 1 : 1000, PhosphoSolutions, Aurora, CO; rabbit anti-NR2A 1 : 2000, AB1555, Millipore, Billerica, MA; rabbit anti-NR2B 1 : 500, AB1557P, Millipore, Billerica, MA). Blots were washed in TBST and incubated with secondary antibodies in 0.1% TBST with 2% non-fat dry milk for 1 hr (horseradish peroxidase-conjugated goat anti-rabbit, 1 : 10,000, Bio-Rad, Hercules, CA). Blots were washed in TBST and TBS and reacted with chemiluminescent reagents (SuperSignal West Pico Chemiluminescent Substrate, ThermoScientific, Rockford, IL). Images were taken with a chemiluminescent ImageQuant LAS4000 Mini (GE Healthcare Life Science, Pittsburgh, PA). The membranes were washed with TBST and stripped with stripping buffer (Restore, ThermoScientific, Rockford, IL). The second immunostaining was the same as described previously except that the primary antibodies were anti-*β*-actin (from mouse, A2228, 1 : 2000 Sigma-Aldrich, Inc., St. Louis, MO, as a loading control for GAD staining) and anti-pan-cadherin (from mouse, C1821, 1 : 1000, Sigma-Aldrich, Inc., St. Louis, MO, as a loading control for receptor staining). The secondary antibody was horseradish peroxidase-conjugated goat anti-mouse (1 : 10000, Bio-Rad, Hercules, CA). The images were analyzed with ImageJ software (NIH, Bethesda, MA). The optical densities of the GAD and GABA receptor bands were normalized to those of the loading control bands (*β*-actin and cadherin) from the same membrane. The relative expression level of each protein from the experimental group was then normalized to the average value of the normal group. 

### 2.9. Statistical Analysis

Statistical comparisons were performed using Sigmastat software (Systat Software Inc., Chicago, IL) and PASW statistic 18 (SPSS Inc., Chicago, IL) and plotted with SigmaPlot software (Systat Software Inc., Chicago, IL) or Matlab (MathWorks Inc. Natick, MA). For within group comparison, paired *t*-tests were used for normally distributed data, whereas Wilcoxon rank-sum tests were used for non-normally distributed data. For between group comparisons, Student's *t*-tests were used for normally distributed data, and Mann-Whitney *U* tests were used for non-normally distributed data. Means are given with standard errors of the mean (±SEM) throughout. 

## 3. Results

Previous studies in our lab demonstrated that auditory function is impaired in XMAC, with broader tuning and less organized tonotopy than in normal animals [20]. Here we investigated the mechanism underlying this auditory impairment, asking whether inhibition is altered in XMAC. For the iontophoresis experiments, forty-one auditory neurons from 7 normal animals and 55 auditory neurons and 24 multisensory neurons from 5 experimental animals were recorded. 

### 3.1. Gabazine Increased Responsiveness to Auditory Stimulation

**Figure 2:**
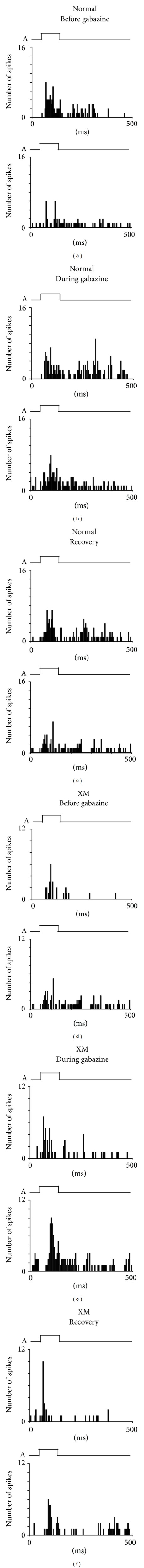
Effects of the GABA_A_ receptor antagonist gabazine on evoked responses to sound. ((a)–(c)) Two examples showing a PSTH of the responses of a single unit to a pure tone at its CF in a normal animal, before, during, and after gabazine application. ((d)–(f)) Two examples showing aPSTH of the responses of a single unit to a pure tone at its CF in a cross-modal animal. “A” in the upper traces of each panel indicates the timing of the auditory stimuli. The duration of each auditory stimulus was 100 ms.

In order to test the hypothesis that altered inhibition contributes to the auditory impairment in XMAC, we examined the effect of gabazine, a GABA_A_ receptor antagonist, on evoked responses to auditory stimulation.  Figure 2 shows the post stimulus time histogram (PSTH) of the responses of a single unit in AC from each group. Stimuli were initiated 50 ms after initiation of data acquisition (Figures 2(a) and 2(d)) so that a baseline, prestimulus firing level could be determined. Evoked responses of each auditory neuron before and after gabazine application (measured by spike counts at 10 dB above threshold, Figure 2) were recorded and compared. As expected for a GABA_A_ receptor antagonist, gabazine application led to an increase in the number of spikes. Response magnitudes returned to the pregabazine level 30 minutes after cessation of drug application (Figures 2(c) and 2(f)). In contrast, injection of a GABA_A_ receptor agonist or an NMDA receptor antagonist decreased responsiveness to auditory stimuli (not shown).

### 3.2. GABA_**A**_ Receptor Blockade Affected Sound Frequency Tuning in XMAC More Than in Normal AC

**Figure 3:**
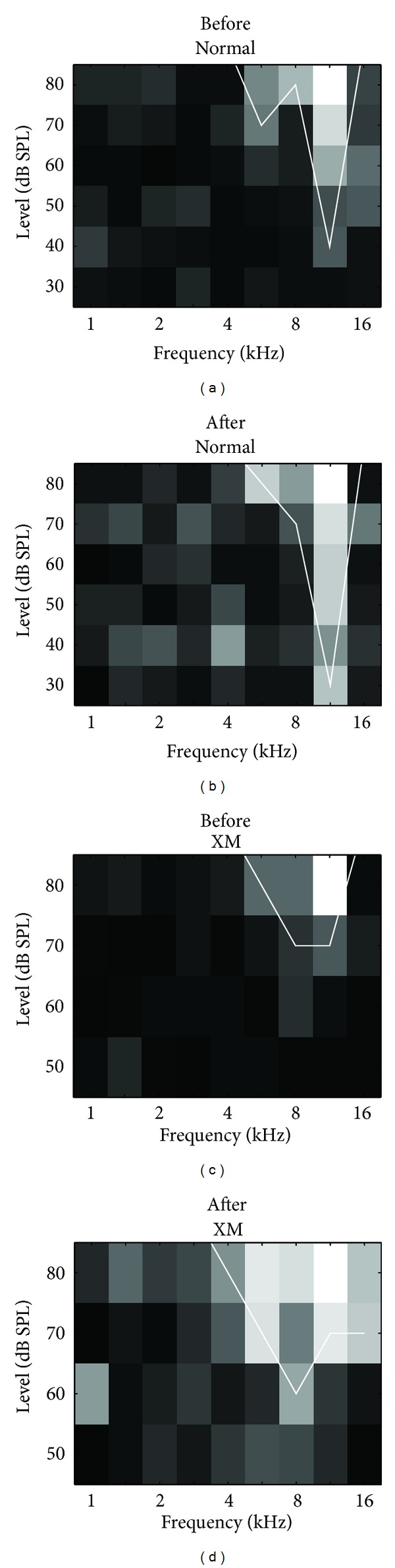
Two examples of the effect of gabazine on sound frequency tuning. (a) An example of an auditory response in a normal animal. (b) Blockade of inhibition by gabazine in the same neuron shown in (a) decreased the threshold of its auditory response. (c) An example of an auditory response from a neuron in XMAC. (d) Blockade of inhibition by gabazine in the same neuron shown in (c) decreased the tuning sharpness and the threshold of the auditory response in this neuron from XMAC.

We reported previously that auditory neurons in XMAC have wider bandwidths and higher thresholds of auditory tuning than auditory neurons in normal AC [20]. We had initially hypothesized that a general loss of inhibition might be responsible for the broader frequency tuning in XMAC compared to normal AC [20]. Alternatively, inhibition in XMAC might be increased as a way to compensate for invasion by ectopic visual inputs and to counteract conflicting activation patterns. To determine how inhibition contributes to sound frequency tuning after cross-modal plasticity, we compared the frequency tuning selectivity (bandwidth) and threshold of auditory responses in normal AC and XMAC. One example before and after gabazine application from each group is shown in Figure 3.

**Figure 4:**
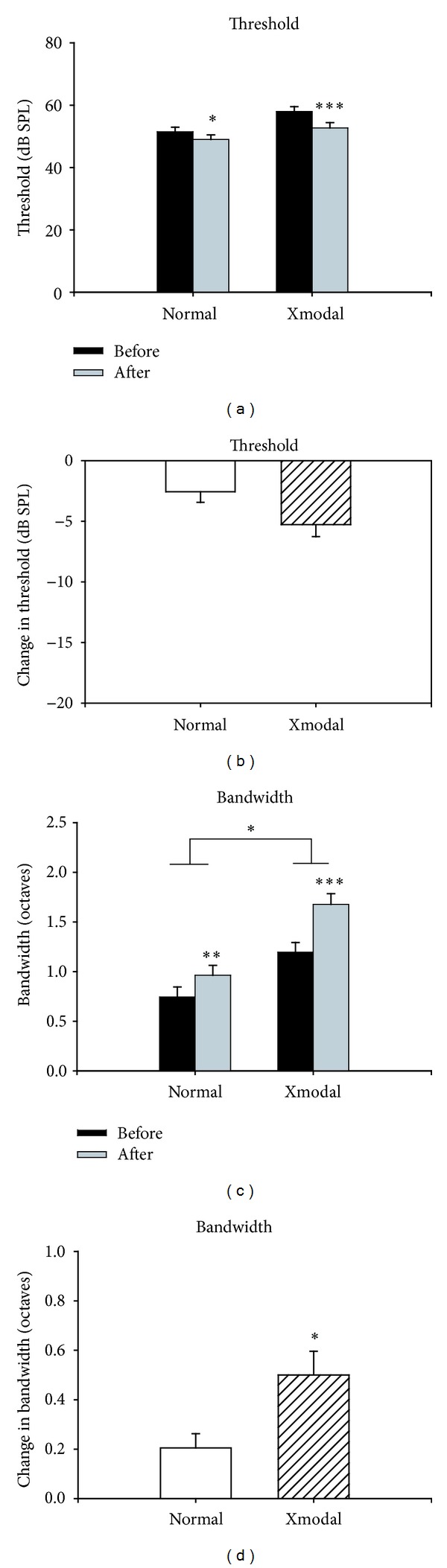
The effects of gabazine on thresholds and bandwidths of auditory tuning curves (population data). (a) Blockade of inhibition by gabazine decreased thresholds in both normal and XMAC. (b) The threshold changes in XMAC were not significantly different from those in the normal AC. (c) Blockade of inhibition by gabazine increased bandwidths in both normal and XMAC. (d) The changes in bandwidth in XMAC were significantly greater than those in the normal AC. *P < 0.05; **P < 0.01; ***P < 0.001.

As expected, the average thresholds of auditory neurons in normal animals decreased slightly after blocking inhibition by gabazine application, from 51.5 ± 1.46 dB to 49.0 ± 1.47 dB (*n* = 41, *P* = 0.03, Wilcoxon rank-sum test, Figure 4(a)). The thresholds of auditory neurons in XMAC were affected by gabazine application, falling from 58.0 ± 1.54 to 52.7 ± 1.69 dB (*n* = 55, *P* < 0.001, Wilcoxon rank-sum test, Figure 4(a)). The decrease in auditory thresholds, however, was not significantly more in XMAC than that in normal AC (normal: −2.7 ± 0.88 dB, XMAC: −5.3 ± 1.00; *P* = 0.1, Mann-Whitney *U* test, Figure 4(b)).

Gabazine application did not produce a shift in characteristic frequency tuning, either in AC neurons in normal animals or in AC neurons of XMAC animals, but gabazine did significantly broaden the bandwidth of the tuning curves in both normal AC (from 0.7 ± 0.10 to 1.0 ± 0.10 octaves at 10 dB above threshold; *n* = 41, *P* < 0.001, Wilcoxon rank-sum test, Figure 4(c)) and in XMAC (from 1.2 ± 0.10 to 1.7 ± 0.11 octaves; *n* = 55, *P* < 0.001, Wilcoxon rank-sum test, Figure 4(c)). The increase in bandwidth was significantly greater in XMAC than that in normal AC (normal: 0.2 ± 0.06 versus XMAC: 0.5 ± 0.10, *P* < 0.05, Mann-Whitney *U* test, Figure 4(d)). Thus, contrary to our initial hypothesis, auditory stimulation evoked stronger inhibition in XMAC than in normal AC. This result suggests that inhibition contributes more to the sharpening of frequency tuning in XMAC than it does in normal AC, and may serve to improve sound frequency tuning after the early cross-modal rewiring has occurred. 

### 3.3. Spontaneous Activity Levels Were Lower in XMAC Than in Normal AC

**Figure 5:**
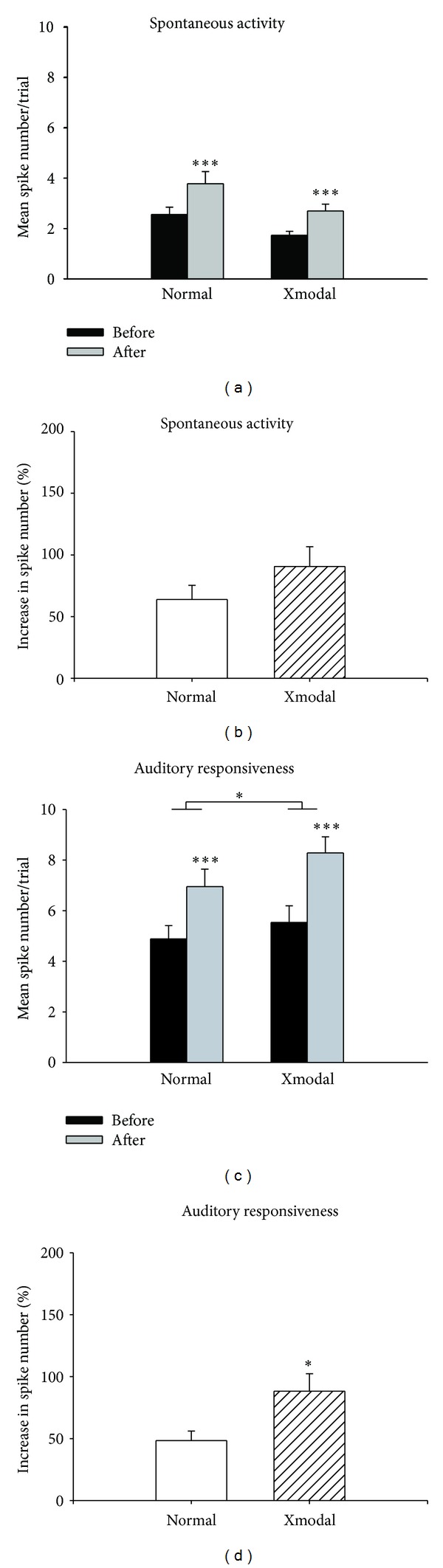
The effects of gabazine on spontaneous activity and auditory responsiveness. (a) Blockade of inhibition by gabazine increased spontaneous activity in both normal and XMAC. (b) The changes in spontaneous activity after gabazine administration in XMAC were not significantly different from those in the normal group. (c) Blockade of inhibition by gabazine increased mean spike numbers per trial in response to auditory stimulation in both normal and XMAC. (d) The changes in responsiveness to sound stimuli after gabazine administration in XMAC were significantly larger than those in the normal group. *P < 0.05; ***P < 0.001.

Increased levels of inhibition could have the effect of an overall depression of activity in auditory cortex. To address this possibility, we compared the spontaneous activity levels in normal AC and XMAC. The spontaneous activity level in XMAC was significantly lower than that in normal AC (normal: 2.6 ± 0.29 spikes per trial; XMAC 1.7 ± 0.15 spikes per trial; Mann-Whitney *U* test, *P* < 0.05). These data suggest that the resting activity level of XMAC is lower than that of normal AC. Blockade of GABA_A_ receptors by gabazine increased spontaneous activity significantly in both normal AC and XMAC (normal before gabazine: 2.6 ± 0.29 spikes per trial versus normal after gabazine: 3.8 ± 0.48, *P* < 0.001; XMAC before: 1.7 ± 0.15 versus XMAC after: 2.7 ± 0.27, *P* < 0.001; Wilcoxon rank-sum test, Figure 5(a)), but the increase was not significantly different between the two groups (normal: 63.8 ± 11.45% versus XMAC: 90.6 ± 15.96%, *P* > 0.05, Mann-Whitney *U* test, Figure 5(b)). This finding argues that the increased inhibition comes into play only during evoked activity and does not have a tonic effect that leads to cortical depression.

### 3.4. GABA_**A**_ Receptor Blockade Affected Responsiveness to Sound in XMAC More Than in Normal AC

To examine the effect of the change in inhibition on evoked activity in XMAC, we also measured the strength of auditory responses before and after blocking inhibition. Before GABA_A_ receptor blockade, there was no significant difference in spike numbers per trial between normal AC and XMAC (normal: 4.9 ± 0.53 spikes per trial; XMAC: 5.5 ± 0.66 spikes per trial; Mann-Whitney *U* test, *P* > 0.05). We found that blockade of GABA_A_ receptors by gabazine increased the average number of spikes per trial significantly in both normal AC (from 4.9 ± 0.53 to 7.0 ± 0.69 spikes, normal before versus normal after, Wilcoxon rank-sum test, *P* < 0.001, *n* = 41, Figure 5(c)) and XMAC (from 5.5 ± 0.66 to 8.3 ± 0.64 spikes, XMAC before versus after, *P* < 0.001; Wilcoxon rank-sum test, *n* = 55, Figure 5(c)). However, this increase in spike number per stimulus was proportionally much greater in the XMAC (88.3 ± 14.1%, *n* = 55 than in the normal group (48.4 ± 7.66%, *n* = 41), *P* < 0.05, Mann-Whitney *U* test, Figure 5(d)). Consistent with the significantly greater change in bandwidth in the XMAC compared to the normal group shown in Figure 4, these results further support the interpretation that auditory neurons in XMAC receive stronger inhibition during sound stimulation than neurons in normal AC. Although this could have the effect of reducing sound responsiveness, it may also improve selectivity for particular auditory objects.

### 3.5. GABA_**A**_ Receptor Blockade Unmasked Visual Responses in Some Auditory-Only Neurons in XMAC

**Figure 6:**
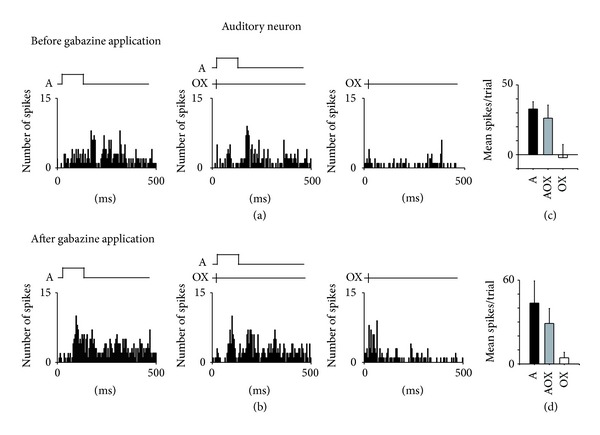
An example of an XMAC neuron's responses to bisensory stimuli summed over 5 trials, before and after blocking inhibition. (a) Before gabazine application, this neuron responded to auditory but not to optic chiasm stimulation. (b) After gabazine application, it responded to both auditory and optic chiasm stimulation. (c) The mean spikes per trial before blockade of inhibition. (d) The mean spikes per trial after blockade of inhibition. Neither facilitation nor depression was found when auditory and optic chiasm stimulation (AOX) were given simultaneously. Note that the neuron began to respond to OX stimulation only after blockade of inhibition. A indicates auditory stimuli. OX indicates optic chiasm stimulation.

Because increased levels of inhibition are evoked by auditory stimulation, we wondered if the same is true upon visual stimulation. Our finding that inhibition is stronger in XMAC than in normal AC suggested to us that auditory and visual inputs may inhibit each other in order for auditory cortex to perform the two different functions within the same region. If this hypothesis is true, then removal of inhibition may unmask responses to the other modality. Therefore, we assayed bisensory responsiveness before and after blockade of inhibition by gabazine. None of the auditory neurons in normal AC responded to visual stimuli under gabazine iontophoresis, but in XMAC, the blockade of inhibition enabled 25.5% of the previously auditory-only neurons (14 out of 55 neurons from 5 XM animals) to respond to electrical stimulation of the optic chiasm (Figures 6(a) and 6(b)). The responses to optic chiasm stimulation were strong, although none of the neurons responded to light stimulation under our conditions. In typical multisensory cortex or multisensory subcortical regions such as the superior colliculus, multisensory neurons show facilitation or depression to multisensory cues [31]. At least under our stimulation conditions, however, no such integration of bisensory stimuli was observed in the XMAC auditory neurons after visual responses were unmasked by GABA (AOX versus A, *P* > 0.05, *t*-test, Figures 6(c) and 6(d)). These results suggest that visual responses in cross-modal auditory cortex are suppressed via a GABA_A_ receptor-dependent process.

### 3.6. GABA_**A**_ Receptor Blockade Did Not Unmask Responses to Light or Cross-Modal Integration in Multisensory Neurons in XMAC

**Figure 7:**
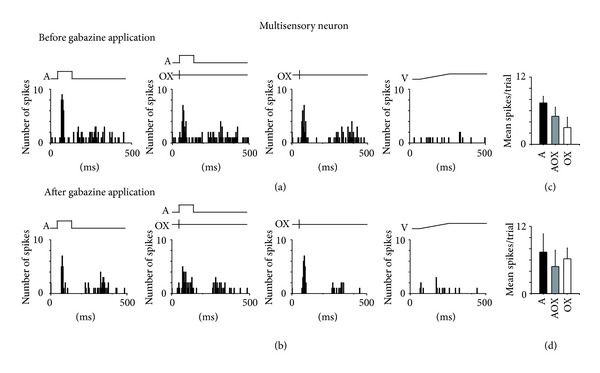
One example of the response of multisensory neurons to auditory and optic chiasm stimulation before and after blockade of inhibition by gabazine. (a) Before gabazine application, this multisensory neuron in XMAC responded to both auditory and optic chiasm stimulation but not to light stimulation. (b) The response type was not changed by gabazine application. (c) The mean spikes per trial before blockade of inhibition. Neither facilitation nor depression was found when auditory and optic chiasm stimulation (AOX) were given together. (d) The mean spikes per trial after blockade of inhibition. Neither facilitation nor depression was found when bimodal stimuli were given. Conventions as in Figure 6.

We reported previously that XMAC contains considerably more multisensory neurons than normal AC [19]. Here we report that the majority of multisensory neurons in XMAC do not exhibit integration with the bisensory stimuli that we employed in this study. Only 8.3% of the recorded multisensory neurons from 5 animals (2 out of 24) showed integration. We reasoned that the lack of integration in XMAC could be caused by GABAergic suppression of responses to the opposing modality. If so, then blockade of inhibition should release facilitation or suppression. We were able to conduct a complete iontophoresis test battery on 8 of the multisensory neurons from these 5 animals in the XMAC group. Contrary to our prediction, none of them showed integration either before or after blockade of inhibition. Before blockade of GABA_A_ receptors, these neurons responded to both sound and optic chiasm stimulation (Figure 7(a)) but none of them showed significant levels of either facilitation or depression when both stimuli were given (AOX versus A or OX; *P* > 0.05, *t*-test, Figure 7(c)). After blockade of inhibition, we still did not observe any significant facilitation or depression of responses during bisensory stimulation (Figures 7(b) and 7(d)) (*P* > 0.05, *t*-test). Furthermore, multisensory neurons that originally responded to both auditory and optic chiasm stimulation did not respond to visual stimulation with light after the removal of inhibition (Figure 7(b)). These results suggest that inhibition does not mask multisensory integration in XMAC. An alternative explanation is that more extensive variation of stimulus parameters (latency, intensity, etc.) may uncover integration. Given the difficulty of recording responsive neurons in during long-term recordings, stimulus repetitions were limited to 5–10 trials for each neuron. Even so, the majority of recorded neurons were lost either during auditory stimulation, visual stimulation, or gabazine application. The optimum stimulus shown to induce maximum integration in previous studies of multisensory cortex [32] was used, so although we did not present all possible stimulus variations, it seems unlikely that we would have missed integration altogether if it existed.

### 3.7. Neurons in Which Gabazine Unmasked Visual Responses Were Spatially Intermixed with Auditory and Multisensory Neurons in XMAC

**Figure 8:**
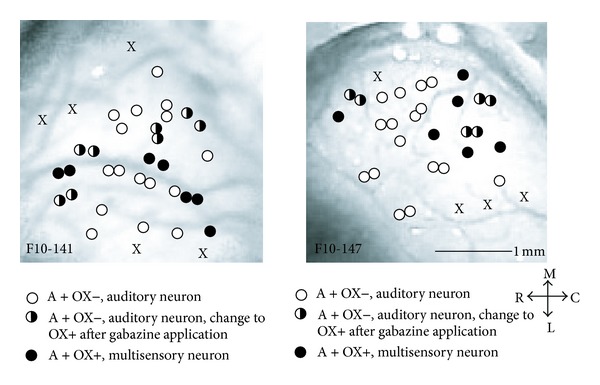
The distribution of neuronal response types before and after application of gabazine. Each panel represents data from one animal. A indicates auditory stimulation. OX indicates optic chiasm stimulation. The + symbols in the legend represent neurons that were responsive to that modality, whereas − symbols represent neurons that were not responsive to that modality. Unmasked visual responses were intermixed with auditory and multisensory neurons. X in the figures indicates a nonresponsive recording site. Scale bar: 1 mm. Arrows at lower right show orientation of the photo. M: medial, L: lateral, R: rostral, and C: caudal.

We reported in previous research that auditory, visual, and multisensory neurons in XMAC are not segregated from each other [19]. The finding of inhibition-masked visual responses in XMAC raised an interesting question concerning their distribution. To determine whether neurons that exhibited unmasking of bisensory capability were a spatially separate population from neurons that did not, we mapped the neuronal responses in XMAC. We found that auditory neurons responding to optic chiasm stimulation after but not before blockade of inhibition were located randomly in XMAC (half white circles, Figure 8), and not selectively in one area. They were found surrounded by or adjacent to either auditory or multisensory neurons (white and black circles, resp., Figure 8). These results show that neurons exhibiting unmasking of bisensory responses were neither segregated nor clustered in their distribution.

### 3.8. The Expression of GABA in Normal and XMAC Neurons

**Figure 9:**
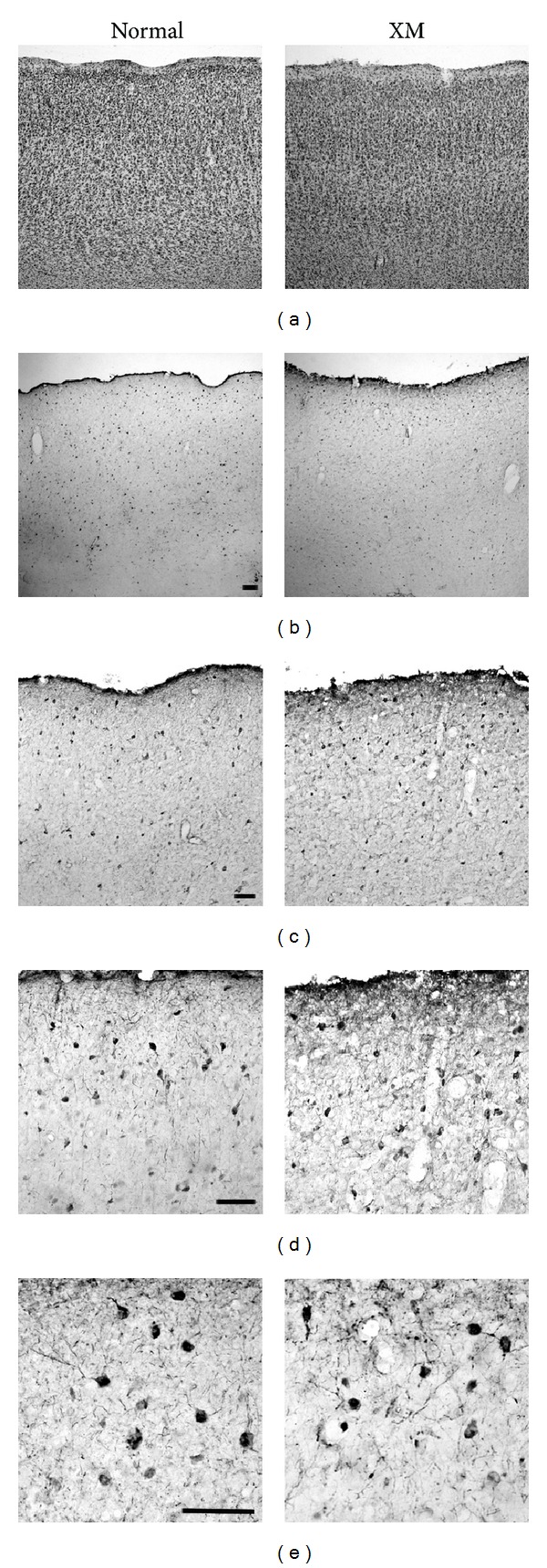
Immunohistochemical staining of GABAergic neurons in auditory cortex of normal and cross-modal animals. (a) Neighboring sections were stained with cresyl echt violet. ((b)–(e)) Increasingly magnified images of the auditory cortex following staining for GABA. Scale bar = 100 *μ*m in all panels.

To determine whether inhibition is increased presynaptically in XMAC, we immunostained GABAergic neurons (see Section 2 for details). In a previous study, we reported that GABAergic neurons in adult ferrets are distributed uniformly across all layers of auditory cortex [28]. Here we report that the laminar distribution of GABAergic neurons in XMAC was similar to that in normal AC (Figure 9). The adjacent Nissl sections indicate that the layer thickness and overall density of neurons in normal and XMAC are similar (Figure 9(a)). We also observed that the overall GABA expression pattern and morphology of GABAergic neurons appeared similar between the normal and the cross-modal group (Figures 9(b)–9(e)). These results suggest that the increase in inhibition found by electrophysiological recordings is not likely achieved by a change in the number or distribution of GABAergic somata. Instead, there may be an increase in the number and/or the strength of existing inhibitory synapses.

### 3.9. The Expression of GAD65 Was Increased in XMAC

**Figure 10:**
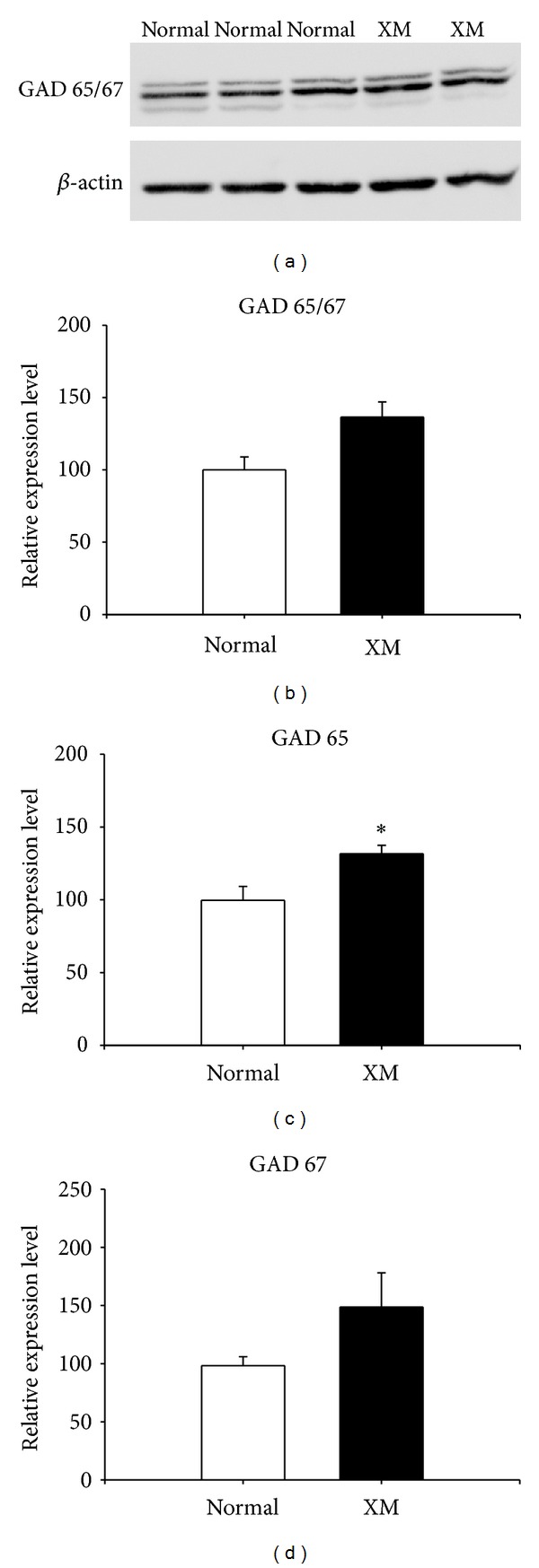
The relative expression of GAD was increased in the cross-modal group. (a) Representative Western blots for GAD and the loading control *β*-actin in the normal and the cross-modal groups. (b) The relative expression level of GAD 65/67. The optical density of GAD was normalized to that of *β*-actin from the same membrane. (c) The relative expression level of GAD 65. There was significantly more expression of GAD in XMAC than in normal AC (∗ represents *P* < 0.05, *t*-test). (d) The relative expression level of GAD 67 was similar between groups.

As an alternative approach to quantifying possible presynaptic changes in inhibition, we applied Western blotting methods to measure the expression of the synthetic enzyme for GABA (GAD). An antibody recognizing both GAD isoforms (GAD 65 and GAD 67) was used. Samples from the same animal were loaded in two or three lanes on the same gel to control for loading variability (Figure 10(a)). The average optical density of GAD staining was normalized to that of *β*-actin staining from the same animal. Although the relative expression levels of GAD 65 and 67 combined did not show significant differences between groups (normal: 100 ± 9.01 versus XMAC: 136.4 ± 10.55, *P* = 0.054, *t*-test, Figure 10(b)), the relative expression level of GAD 65 alone in XMAC was significantly higher than that in normal AC (normal: 99.6 ± 9.55 versus XMAC: 131.6 ± 5.82, *P* < 0.05, *t*-test, Figure 10(c)). The relative expression level of GAD 67 in XMAC (151.6 ± 26.09) was not significantly different from that in normal AC (101.8 ± 7.37, *P* > 0.05, *t*-test, Figure 10(d)). These results suggest that inhibition in XMAC increases at least in part at the presynaptic side of the synapse through an increase in GABA content at synapses. Although both isoforms of GAD (GAD 65 and GAD 67) are present in most GABAergic neurons in the brain, GAD 67 is more abundant in cell bodies, whereas GAD 65 is more prominent in axon terminals [33, 34]; perhaps explaining why a change in proportion of GABA-ir cell bodies was not seen (cf. Figure 9). Because the majority of GAD 65 exists in an inactive form, its localization at axon terminals is convenient for synthesis of GABA when synaptic activity requires it [34]. Our finding that GAD 65 increases significantly supports the electrophysiological data showing that inhibition is increased at synapses in XMAC.

### 3.10. The Expression Level of GABA_**A**_ Receptors Was Not Altered in XMAC

**Figure 11:**
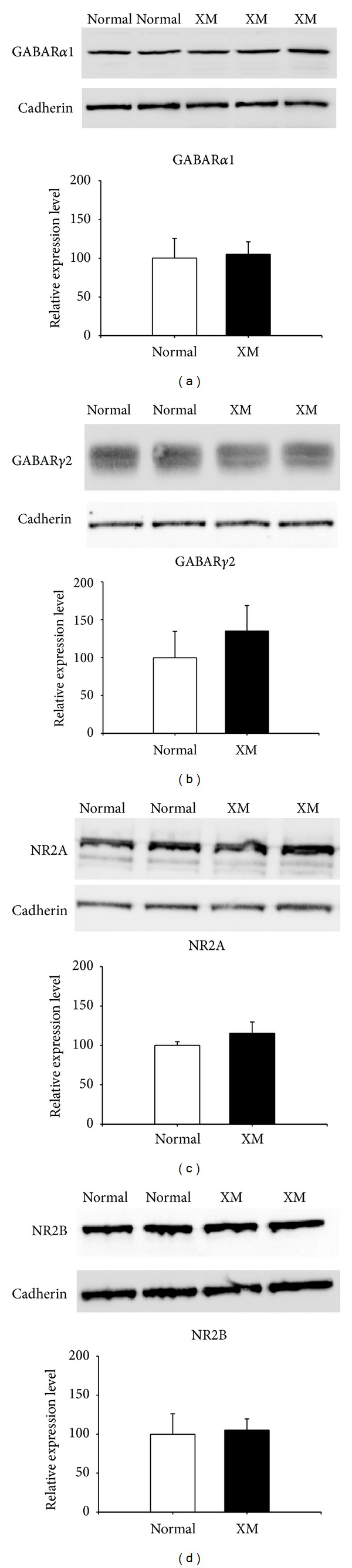
The relative receptor expression levels were not changed in the cross-modal group compared to the normal group. (a) Representative Western blots for GABA_A_R*α*1 subunit protein and the corresponding optical density measurements. (b) Representative Western blotting for GABA_A_R*γ*2 subunit protein and the corresponding optical density measurements. (c) Representative Western blotting for NMDAR NR2A subunit protein and the corresponding optical density measurements. (d) Representative Western blots for NMDAR NR2B subunit protein and the corresponding optical density measurements.

To determine whether there were postsynaptic changes in inhibition and excitation in XMAC, we applied Western blotting methods to quantify the expression levels of GABA_A_ receptors and NMDA receptors. GABA_A_R*α*1 and GABA_A_R*γ*2, two of the most prevalent subunits of GABA_A_ receptors, were examined. NR2A and NR2B, the two plasticity-related subunits of NMDA receptors, were also examined. The average optical density of labeled receptors was normalized to that of cadherin from the same animal to provide a loading control. We did not find any differences in receptor expression levels between the normal and the cross-modal groups. The relative expression level of GABA_A_R*α*1 (105.1 ± 16.09) in the cross-modal group was not significantly different from that in the normal group (100.1 ± 25.49, *P* > 0.05, *t*-test, Figure 11(a)). In addition, no significant differences were found between groups in the expression of GABA_A_R*γ*2 (normal: 99.8 ± 34.80 versus XMAC: 134.9 ± 33.71, *P* > 0.05, *t*-test, Figure 11(b)). The relative expression levels of NR2A (115.3 ± 14.34) and NR2B (105.1 ± 14.31) in XMAC were also not significantly different from those in normal AC (NR2A: 100 ± 4.61, *P* > 0.05, *t*-test, Figure 11(c); NR2B: 99.8 ± 26.38, *P* > 0.05, *t*-test, Figure 11(d)). These results suggest that the overall expression levels of inhibitory and excitatory neurotransmitter receptors do not change in auditory cortex as a result of invasion by visual inputs.

## 4. Discussion 

The main finding in this study was that neurons in auditory cortex that had been rewired at birth with ectopic visual inputs (XMAC) received stronger inhibition than neurons in normal auditory cortex. Blockade of GABA_A_R in XMAC increased auditory responses to a proportionally greater extent in XMAC neurons than in normal AC neurons. The expression level of GAD protein but not of GABA_A_ receptors was increased in XMAC compared to normal AC. This result implies that the broadened auditory tuning found in XMAC does not result from decreased lateral inhibition from neighboring frequency bands. 

The increased inhibition in XMAC suggests a role for inhibitory plasticity in XMAC after the period of recovery. For example, we found that blockade of inhibition unmasked responses to stimulation by the other modality in some neurons. This finding argues that the increased inhibition in XMAC plays a role in suppressing responses to the opposing modality during bisensory stimulation. This interpretation is supported by a study on deaf people with cochlear implants, which showed that a visual-dominant bias occurs in audiovisual integration of speech cues, whereas normal hearing individuals exhibit a balance in the importance of cues from the two modalities [35]. Here we provide a possible mechanism through which increased inhibition may improve the functioning of the auditory cortex for its original task of sound perception while sacrificing multisensory function. Because the multisensory neurons that are created as a result of the visual inputs could cause perceptual confusion or disrupt normal auditory perception, the increase in intersensory inhibition may allow the auditory cortex to function despite the anomalous input resulting from the early manipulations. These results provide important information about possible barriers to rehabilitation from sensory dysfunction, particularly when cross-modal plasticity occurs. 

### 4.1. Contribution of GABAergic Inhibition to Auditory Tuning after Invasion by Cross-Modal Inputs

In a previous study, we found broader auditory tuning in XMAC than in normal AC. This could occur through a homeostatic decrease of inhibition in XMAC, as occurs in sensory-deprived and deafferented animals (see [1], for review). During development, refinement of synaptic connections involves suppression of some responses by increasing inhibition [36]. Following reductions in afferent drive, through brain damage, sensory deprivation, or deafferentation, the strength of inhibition is decreased to compensate for the loss of inputs, thus maintaining a balance between excitation and inhibition ([37], see [38, 39], for review, [40]). In auditory and somatosensory cortex, deafferentation can induce an immediate expansion of adjacent inputs into the affected area of the cortex [41–44] that results from a loss of inhibition [45–48]. Similarly, in visual cortex, visual deprivation leads to broader orientation tuning and larger receptive fields [49, 50]. This change is caused at least in part by a loss of inhibition [51]. Then how can the increased level of inhibition in XMAC be explained? Cross-modal plasticity in our model involves adding ectopic visual inputs and thus does not represent the degree of sensory loss seen in deafness. The addition of the visual inputs may prevent a loss of inhibition that could result from deafferentation of MGN. 

 The decrease of inhibition within one modality after loss of inputs can be transient. It has been found that GABA expression in sensorimotor cortex is reduced quickly after forearm nerve block [52] but can recover to normal levels in adult sensory-deprived or deafferented animals. For example, the expression of GABA in inferior colliculus is decreased after unilateral cochlear lesions but returns to normal levels one month later [53]. We found previously that neonatal damage to auditory midbrain in the absence of visual invasion did not significantly alter auditory tuning and tonotopy [20], suggesting that thalamocortically activated lateral inhibition may recover to normal levels after IC lesions. These results thus suggest that the broadening of auditory tuning results from visual invasion and not from deafferentation. After transient changes following damage to sensory pathways, it is important to reestablish receptive fields and excitability levels. Increasing inhibition may play an important role during this process [54]. Furthermore, loss of sensory inputs from one modality can lead to enhanced perception to the other stimuli [3, 55]. This cross-modal improvement might result from an increase in inhibition. In mice that have olfactory deficits, whisking responses in the barrel cortex are upregulated. With this upregulation, the number of GABAergic neurons is increased [56]. We also found increased inhibition in the auditory cortex after invasion by cross-modal inputs. After recovery from visual invasion, on the one hand, XMAC is reorganized to manage responses to both auditory and visual stimuli. Because auditory and visual neurons are not segregated from each other [19], responses to a single modality may be disturbed by responses to the other modality. Mutual inhibition could be increased in XMAC in order to suppress neighboring firing that may represent a different modality. On the other hand, sprouting of residual auditory inputs to the cortex could contribute to the broader tuning, whereas a reduction in excitatory auditory inputs to XMAC is a possible explanation for the higher auditory thresholds. It seems plausible that the increased inhibition helps to compensate for the decrease in sharpness of tuning that occurs with cross-modal plasticity, and that without it, tuning would be even more compromised. At any rate, the reorganization of XMAC seems to involve a different mechanism than does cortical reorganization after single modality deprivation or deafferentation. 

### 4.2. Possible Mechanism Underlying Impaired Auditory Function after Cross-Modal Plasticity

Our finding that loss of inhibition is not responsible for broader than normal tuning in XMAC may be explained by the role of lateral inhibition in refinement of auditory tuning during development. Local inhibition shapes sound frequency tuning in auditory cortex [57–60]. Using whole cell recording in auditory cortex, it has been argued that the inhibitory component of auditory receptive fields shows tuning that is similar to the tuning of the excitatory component [61–63], suggesting that refinement of auditory receptive fields may not require feed-forward lateral inhibition. Although intracortical inhibition is necessary for construction of auditory receptive fields [64, 65], refinement of auditory receptive fields relies more on the maturation of excitatory inputs [66] and a balance between excitation and inhibition, rather than on inhibition itself [67, 68]. Here we found that inhibition was increased in XMAC after recovery from neonatal midbrain lesions, despite the fact that auditory tuning was broader in XMAC than in normal AC. Because refinement of auditory receptive fields could depend on narrowing of the axonal projections from excitatory thalamocortical inputs, the widened auditory tuning in XMAC could result from sprouting of auditory thalamocortical afferents after lesion of the inferior colliculi. Our data suggest that excitatory input may play a role in constructing auditory properties during recovery from midbrain damage.

### 4.3. Multisensory Processing in Primary Sensory Areas

Several recent studies have demonstrated multisensory inputs to primary sensory areas (e.g., [25, 69, 70]), arguing that primary sensory cortex is not “primary” in the sense of receiving only unimodal thalamic input [71]. Bizley and colleagues found visual responses in auditory neurons at the lateral edges of A1, using a more sensitive method that picked up subthreshold activity. Multisensory integration (defined as facilitation or suppression caused by adding the second modality) is a defining characteristic of multisensory neurons in most multisensory cortical and subcortical regions [32, 72]. In cortex receiving bimodal input integration does not necessarily occur, but subthreshold multisensory integration can be released in some neurons by the removal of inhibition [73]. Here we found that after application of GABA receptor antagonists, some auditory neurons were capable of responding to visual stimuli. However, no integration (neither facilitation nor suppression) of these multisensory responses was revealed before or after unmasking. When responses to multiple modalities are forced to coexist in XMAC, multisensory integration would likely interfere with rather than improve sensory perception, especially for auditory-dominant multisensory neurons that are tuned to pure tones. It has been shown that multisensory neurons in superior colliculus lack integration after animals were raised in the dark [74], whereas previously “silent” sensory input channels can be revealed by cross-modal exposure [75]. Our findings provide a possible explanation for these results. Inhibitory plasticity could be the mechanism underlying both the lack of integration and the reopening of silent channels. Overall, our results suggest that injury-induced convergence of multisensory inputs to one primary cortical area can create neurons responding to different modalities, but multisensory integration is suppressed, much like underdeveloped multisensory neurons found in sensory-deprived animals [74]. Further research on cross-modal animals with sensory exposure would shed more light on this question.

## 5. Conclusions

Taken together, our results suggest that an increase in inhibition contributes to recovery from neonatal deafferentation that impacts multiple modalities. Recently, it was found that inhibition is increased in the peri-infarct zone in mice after stroke. The researchers applied the GABA_A_ benzodiazepine antagonist specific for the *α*5-subunit in a mouse stroke model and found that forelimb and hindlimb control was recovered, suggesting that pharmacological treatment is a possible way to counteract abnormal function after maladaptive plasticity [76]. Our findings of increased inhibition in XMAC may therefore be informative for the design of rehabilitative strategies or pharmacological interventions for patients who have brain damage, sensory/motor dysfunction, or sensory deprivation.
